# Adaptive Fusion of LiDAR Features for 3D Object Detection in Autonomous Driving

**DOI:** 10.3390/s25133865

**Published:** 2025-06-21

**Authors:** Mingrui Wang, Dongjie Li, Josep R. Casas, Javier Ruiz-Hidalgo

**Affiliations:** 1 Image Processing Group, TSC Department, Polytechnic University of Catalonia (UPC), 08034 Barcelona, Spain; mingrui.wang@upc.edu (M.W.); josep.ramon.casas@upc.edu (J.R.C.); 2 Key Laboratory of Advanced Manufacturing and Intelligent Technology Ministry of Education, School of Mechanical and Power Engineering, Harbin University of Science and Technology, Harbin 150080, China; dongjieli@hrbust.edu.cn

**Keywords:** autonomous driving, cooperative perception, data fusion, object detection, LiDAR system, sensor fusion

## Abstract

In the field of autonomous driving, cooperative perception through vehicle-to-vehicle communication significantly enhances environmental understanding by leveraging multi-sensor data, including LiDAR, cameras, and radar. However, traditional early or late fusion methods face challenges such as high bandwidth and computational resources, which make it difficult to balance data transmission efficiency with the accuracy of perception of the surrounding environment, especially for the detection of smaller objects such as pedestrians. To address these challenges, this paper proposes a novel cooperative perception framework based on two-stage intermediate-level sensor feature fusion specifically designed for complex traffic scenarios where pedestrians and vehicles coexist. In such scenarios, the model demonstrates superior performance in detecting small objects like pedestrians compared to mainstream perception methods while also improving the cooperative perception accuracy for medium and large objects such as vehicles. Furthermore, to thoroughly validate the reliability of the proposed model, we conducted both qualitative and quantitative experiments on mainstream simulated and real-world datasets. The experimental results demonstrate that our approach outperforms state-of-the-art perception models in terms of mAP, achieving up to a 4.1% improvement in vehicle detection accuracy and a remarkable 29.2% enhancement in pedestrian detection accuracy.

## 1. Introduction

With the rapid development of autonomous driving technologies, self-driving cars [1,2,3] are gradually entering everyday life and becoming an essential component of future intelligent transportation systems. Emerging paradigms such as intelligent connected vehicles, vehicle–road cooperation, vehicular networks, and smart mobility are reshaping the automotive industry and accelerating the advancement of modern transportation systems [4,5]. Among the core technologies enabling autonomous driving, LiDAR point clouds play a crucial role in environmental perception by supporting accurate 3D object detection and precise localization in real-world scenarios. However, conventional 3D object detection algorithms often struggle in complex environments, particularly with small or occluded objects, resulting in reduced detection accuracy.

In recent years, the reliability of vehicle-to-vehicle (V2V) collaborative perception algorithms [6,7,8] has significantly improved, largely driven by advancements in neural network architectures and the intelligent fusion of multi-modal sensor data, such as LiDAR, images, and radar. Compared to single-vehicle perception, V2V collaboration allows multiple connected autonomous vehicles (CAVs) to share and integrate complementary sensory information across different viewpoints. This collaborative approach addresses limitations caused by occlusion and field-of-view constraints, improving global perception performance in dynamic traffic environments. Furthermore, sophisticated feature fusion strategies have demonstrated strong robustness in recognizing objects, even under conditions of adverse weather and congested traffic [9].

Current cooperative perception fusion methods among vehicles are mainly categorized into three types: early fusion, late fusion, and intermediate fusion [10]. These fusion strategies differ significantly in terms of sensor data redundancy, total data volume, and the effectiveness of fusion results. In early fusion approaches, raw sensor data from different connected autonomous vehicles (CAVs) are aggregated to build a global driving environment perspective [11]. Although such methods have demonstrated remarkable performance in addressing occlusion and field-of-view limitations inherent in single-vehicle perception, they come at the cost of high communication resource demands. The heavy transmission load and large volume of shared data can lead to communication network congestion and latency, thereby affecting the usability and stability of the models in real-world applications. Under the premise of limited communication bandwidth, early fusion becomes increasingly impractical and inefficient in complex traffic scenarios with large data volumes, ultimately constraining the effectiveness of perception performance.

Late sensor data fusion methods (late fusion) [12,13] achieve global collaborative perception by merging the perception results independently generated by individual vehicles. Compared with early fusion methods, late fusion only requires the transmission of processed detection results, allowing each vehicle to independently process its own sensor data, then perform unified data fusion afterward. This approach facilitates system modularization and enables autonomous detection and decision-making by individual vehicles, thereby reducing dependence on real-time, high-bandwidth communication. However, current collaborative perception approaches based on late fusion rely heavily on the local perception results of individual vehicles rather than aggregated global data. If all participating vehicles were able to share sensor data, more statistically meaningful data processing would be possible, leading to more accurate detection and tracking of objects in the environment. Therefore, to achieve optimal overall performance, it is essential to consider the global nature of sensor data within the perception range and perform thorough and effective fusion accordingly.

Intermediate-level feature fusion [14,15,16] refers to the extraction of intermediate feature maps within each connected autonomous vehicle (CAV) using a predictive model, followed by the filtering and aggregation of these features in the intermediate feature space. Unlike early fusion methods that require the transmission of raw sensor data, intermediate fusion techniques only transmit these processed feature maps to other CAVs or to edge computing servers in the infrastructure. These intermediate features are then fused and decoded by each autonomous vehicle to generate final perception results. As a compromise in V2V cooperative perception strategies, intermediate-level fusion has the potential to significantly reduce inter-vehicle communication bandwidth requirements compared to early fusion while also demonstrating strong performance in enhancing perception accuracy [17,18]. Compared to late fusion methods, this approach avoids the limitations caused by reliance on local perception results from individual vehicles by efficiently compressing representative global information of the environment into intermediate features, thereby achieving a better trade-off between transmission efficiency and perceptual effectiveness.

Based on the aforementioned challenges in perception accuracy and bandwidth constraints, this paper introduces a novel collaborative perception framework through an efficient Two-Stage Intermediate-level Feature Fusion (TS-IFF) strategy. The proposed framework focuses on the effective aggregation of multi-scale features while maintaining low communication overhead. By integrating a dynamic fusion model, TS-IFF enables adaptive and robust feature combination, leading to enhanced 3D object detection performance in complex traffic scenarios. The key contributions of this work are summarized as follows:

1. We design a collaborative perception architecture based on a novel TS-IFF framework that hierarchically fuses intermediate features to balance perception accuracy and communication bandwidth.
2. To enhance the detection of small and occluded objects, we propose a dual-branch fusion strategy that combines high-resolution pseudo-image features with contextual intermediate-level features for richer semantic representation.
3. We introduce a Dynamic Weight Learning Mechanism (DWLM) to learn fusion weights for different feature types and develop an Adaptive Feature Selection Module(AFSM) to selectively aggregate the most informative components during the fusion process.

## 2. Related Work

### 2.1. 3D Object Detection

Accurate object perception is crucial for safety in autonomous driving. The current leading 3D object detection models primarily use deep learning on 3D point clouds, a key area in 3D object detection [19]. These models directly process raw point-cloud data to reduce information loss and utilize 3D geometry. PointNet [20] achieves end-to-end recognition through point-wise feature extraction and global pooling. To improve local geometric modeling, DGCNN [21] introduces dynamic compositional convolution via a graph convolutional network that enables point adjacency adjustments. Transformer-based models like Point Transformer [22] further improve accuracy by integrating global and local information with self-attention. These methods refine feature extraction and point-cloud representation by utilizing sparse structures to balance computational efficiency and information preservation. Techniques such as the use of anchor points [23] and center strategies [24,25] improve accuracy and real-time performance. In addition, BirdNet+ adopts a BEV-based approach using Faster R-CNN to directly predict 3D object boxes, achieving competitive accuracy and efficiency on the KITTI and nuScenes datasets [26]. These results highlight the effectiveness of compact BEV representations for real-time 3D detection across diverse environments.

While using point clouds preserves 3D information, data sparsity, especially at long distances or in complex environments, poses a challenge for feature extraction. Sparse distributions hinder the network’s ability to generate accurate feature representations, impacting detection accuracy and robustness. Solutions such as voxelization and bird’s-eye-view projection are used to improve the geometry of LiDAR point clouds. For example, VoxelNet [27] encodes voxel features with PointNet++ [28] and applies a region proposal network, while SECOND [29] boosts performance with sparse convolution. CenterPoint refines the backbone outputs into feature maps and predicts object centers from heat maps. However, in real urban driving environments (with obstacles such as buildings, trees, and traffic signs), individual vehicle perception from a single point of view is prone to occlusion, leading to information loss or misclassification [30,31]. Therefore, the integration of sensor data from multiple CAVs is a promising approach to improve 3D object recognition in real traffic conditions.

### 2.2. Cooperative Perception

To overcome the limitations of single-vehicle perception in complex environments, cooperative perception with multiple AVs has become widely adopted [32]. LiDAR and camera data from surrounding vehicles or roadside infrastructures are important sources for sharing observations in cooperative perception. Intermediate-level feature fusion provides a balance between performance and efficiency by effectively merging features from nearby vehicles. F-Cooper [33], the first intermediate collaborative perception system, uses feature-level fusion by taking the maximum value of overlapping regions. Based on this, CoFF [34] addresses F-Cooper’s disregard for low-confidence features. Attention mechanisms, including visual transformers such as V2X-ViT [35] and CoBEVT [36], further improve the relationships between features. In high-resolution detection, MSwin [37] captures spatial interactions over large distances, while AttFusion [38] applies self-attention to specific spatial locations. AdaFusion [39] introduces adaptive fusion models with trainable neural networks. CORE [12] reconstructs incomplete scenes perceived by a single vehicle into a comprehensive view using a compressor, an attention module, and a reconstruction module. However, most existing cooperative perception methods focus on merging a single type of intermediate features, overlooking the benefits of combining multiple feature types. Therefore, we propose a novel perceptual model that integrates intermediate features across different stages.

## 3. Overall Network Architecture

**Figure 1:**
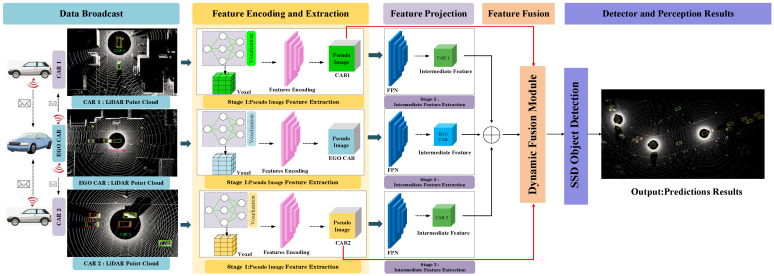
Overview of a collaborative perception framework based on Two-Stage Intermediate-Level Feature Fusion (TS-IFF). The system fuses LiDAR data from multiple autonomous vehicles, as demonstrated here with three collaborating CAVs. Each point-cloud datum is voxelized to generate voxel-level pseudo-images. Pseudo-images are passed through a feature extraction layer (FPN) to extract corresponding intermediate features. Our proposed fusion module integrates the features from both stages and from all CAVs. A final single-shot detector (SSD) produces the detection results. Note that ⊕ represents concatenation. In the visual representation, differently colored arrows illustrate data flow, while bold red lines highlight connections related to the fusion of pseudo-image features.

The overall structure of the network is shown in Figure 1, which can be divided into the following five parts:

1. *Data Generation*: Following the methodology of [38], a spatial graph is first constructed to effectively integrate the relative poses and geographic locations of each connected and autonomous vehicle (CAV), enabling reliable sharing of localization information across the network. Then, the LiDAR data from each CAV in the network is projected onto a unified reference self-coordinate plane for alignment. The aligned point cloud features are broadcast to all participating CAVs in the cooperative perception system, forming the initial stage of inter-vehicle feature interaction and preparing for the next phase of point-cloud encoding and extraction.
2. *Feature Encoding and Extraction*: Each CAV processes the received point-cloud features using a combination of a Voxel Feature Encoding (VFE) module and a PointPillar-based feature extraction network. The VFE module generates voxelized features with different resolutions, resulting in pseudo-images. These pseudo-images from different viewpoints are handled in two ways: (1) they are broadcast to a central dynamic fusion module to be integrated with the intermediate-level features from the ego CAV, and (2) they are retained locally to enable the extraction of intermediate features by the CAV itself. This stage enables distributed local encoding and centralized fusion interactions. (Section 3.1).
3. *Feature Projection*: A Feature Pyramid Network (FPN) [40] is used to extract intermediate features from the pseudo-images. The network follows a top-down structure, first extracting semantic features through downsampling blocks with 2D convolution, batch normalization, and ReLU activation, then processing them through upsampling and lateral connections to generate multi-scale intermediate-level features. The projected features are unified in channel dimension, concatenated, and transmitted to the feature fusion module. Through the Dynamic Weight Learning Mechanism (DWLM), local pseudo-image features are adaptively fused, enabling fine-grained feature interaction across multiple CAVs. (Section 3.2).
4. *Feature Fusion*: All pseudo-images and intermediate-level features from participating CAVs are aggregated via the proposed dynamic fusion strategy. The system performs cross-agent feature integration by assigning adaptive weights to each feature channel based on its contribution. The Adaptive Feature Selection Module (AFSM) refines the joint features further to ensure that the final representation maintains discriminative cues from both local and shared contexts. (Section 3.3).
5. *Object Detector*: Finally, a standard Single-Shot Detector (SSD) network [41] is applied to the fused intermediate features to classify 3D objects and regress their locations. The end-to-end detection result is enhanced by the preceding multi-agent collaborative encoding and fusion steps.

### 3.1. Feature Encoding and Extraction

We used the VFE module from [27] to project the original point cloud onto the bird’s-eye-view (BEV) plane. This process involves calculating the 3D (X,Y,Z) indices of each point and transforming point-level features into voxel-level features, represented as a four-dimensional tensor, i.e., $V\in R^{C\times H\times W\times Z}$. To further process these features, we integrated the PointPillars method [31], which reorganizes the tensor by collapsing the *Z* dimension through scatter operations and pooling, resulting in a columnar structure. Essentially, PointPillars treats vertical columns (pillars) on the BEV plane as spatial bins, aggregating and encoding features from all points within the same pillar to create a dense 2D pseudo-image ($F_{p}\in R^{C\times H\times W}$) that effectively represents the 3D point cloud.

The pseudo-image generated by PointPillars and the intermediate features extracted by an FPN differ fundamentally in structure and representation. PointPillars converts the raw point cloud into a 2D pseudo-image by dividing the space into vertical columns and applying PointNet to each pillar. This process compresses the 3D spatial information into a BEV feature map, emphasizing efficiency and regular grid alignment suitable for 2D convolution.

In contrast, the intermediate features extracted via an FPN operate on multi-scale hierarchical representations of the input, often preserving richer semantic and spatial context across resolutions. When applied to point-cloud data (e.g., using sparse convolution backbones), FPN features retain more local geometric details and cross-scale dependencies, which are essential for detecting objects of varying sizes and densities in 3D space. In summary, while PointPillars emphasizes structured efficiency via BEV pseudo-images, FPN-derived features focus on multi-level abstraction and geometric richness, often at a higher computational cost but with improved accuracy in complex scenes.

**Figure 2:**
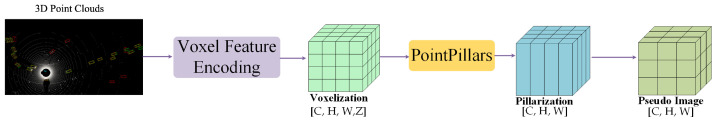
Schematic representation of point-cloud feature encoding. When using a given voxel size, the voxelization of the point cloud leads to voxel features of size [C,H,W,Z]. After pillarization, the *Z* dimension is collapsed. If a sample or pillar has too little data to populate the tensor, zero-padding is applied. Through this encoding process, high-resolution pseudo-images of the point cloud can be generated, serving as the input for subsequent feature extraction and fusion.

To optimize the input resolution of the pseudo-image, we adjusted the voxel size, experimenting with values ranging from 0.4 m down to 0.12 m, which controls the dimensions [C,H,W] of the pseudo-image. Our experiments indicate that higher pseudo-image resolution improves the performance of downstream feature fusion-based object detection tasks. However, when extracting intermediate-level features from the pseudo-image using the FPN [40], the downsampling modules produce intermediate features with a fixed output resolution. Thus, the spatial resolution of the intermediate FPN features remains unchanged, despite variations in the resolution of the input pseudo-image. A schematic diagram illustrating the point-cloud feature encoding process is provided in Figure 2.

### 3.2. Feature Fusion and Object Detection

Pseudo-images generated from raw point-cloud data effectively capture the spatial structure of the environment, preserving detailed geometric information, while intermediate-level features extracted from point clouds provide rich multi-scale contextual semantics. In this paper, we propose a novel collaborative perception fusion strategy that adaptively integrates these two types of features, fully exploiting their complementary strengths in feature representation. The fused feature maps significantly enhance the accuracy of 3D object detection, particularly in complex environments involving small and distant targets. By incorporating both pseudo-images and intermediate-level features, the proposed fusion strategy diversifies the feature representation and improves detection robustness, outperforming other methods that rely solely on intermediate features.

The fusion process is carried out in two stages. In the first stage, a set of fusion weights (W) is generated by a DWLM, which dynamically adjusts and optimizes the contributions of the different pseudo-images and intermediate features based on their relevance. In the second stage, inspired by the structure of the SENet module [42], we propose the AFSM to define feature mappings by selecting and fusing channel information. These fusion weights are used to effectively integrate and refine all pseudo-images and intermediate features from all cooperating CAVs. This two-stage approach ensures optimal spatial and semantic fusion of features and significantly improves the model’s ability to perform accurate object detection in diverse and challenging driving scenarios.

### 3.3. Dynamic Weight Learning Mechanism

**Figure 3:**
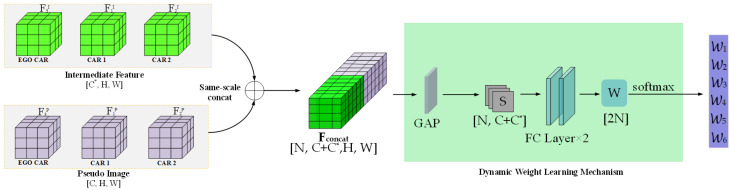
Dynamic weight learning mechanism: the intermediate point-cloud features ($F_{1}^{I}$,$F_{2}^{I}$,$F_{3}^{I}$) of three autonomous vehicles (car1, car2, and the ego vehicle) and their pseudo-image features ($F_{1}^{P}$,$F_{2}^{P}$,$F_{3}^{P}$) are combined as input features. A cascade operation generates concatenated features ($F_{\mathrm{concat}}$, where *W* and *H* are the feature width and height, *C* and $C^{*}$ are the channel numbers of different modality features, *N* is the number of fused CAVs, S is the feature vector, and W is the feature fusion weight).

The DWLM is shown in Figure 3. Before fusion, we concatenate pseudo-images ($F_{n}^{P}$) with intermediate-level features ($F_{n}^{I}$) (where *n* identifies the CAV). Before concatenation, intermediate-level features are upsampled to match the resolution of the pseudo-images. The final concatenated feature corresponds to a tensor ($F_{\mathrm{concat}}\in R^{N\times (C+C^{*})\times H\times W}$, where *N* represents the total number of fused CAVs and *C* and $C^{*}$ denote the channel numbers of the pseudo-images and intermediate-level features, respectively). Subsequently, global average pooling is applied to $F_{\mathrm{concat}}$ to reduce the dimensionality in the last two dimensions, resulting in the feature vector ($S\in R^{N\times (C+C^{*})}$).

Adaptive fusion weights are learned based on the channel-wise aggregated statistics, allowing the network to emphasize more informative modalities or feature levels. This vector (S) is passed through two fully connected layers of the same dimension to learn the importance of each channel, thereby producing the fusion weight vector ($W\in R^{2N}$). Certain channels may focus more on edge structures, dense regions, or local geometric features, which are differently captured by pseudo-images and intermediate features. Thus, the network is trained to automatically determine the appropriate balance between them, depending on their semantic richness and discriminative capacity. The final weight vector (W) is divided into two *N*-dimensional sub-vectors, controlling the fusion ratio of pseudo-image and intermediate-level features, respectively. To ensure training stability of the weight learner, softmax normalization is applied to W, yielding 2N adaptive fusion weights. These weights control the contribution ratios of the pseudo-image features and intermediate-level features in the final fused representation. Finally, the input features are linearly weighted and fused based on the learned fusion weights. This approach enables efficient and robust integration of features across modalities and CAVs.

### 3.4. Adaptive Feature Selection Module

**Figure 4:**
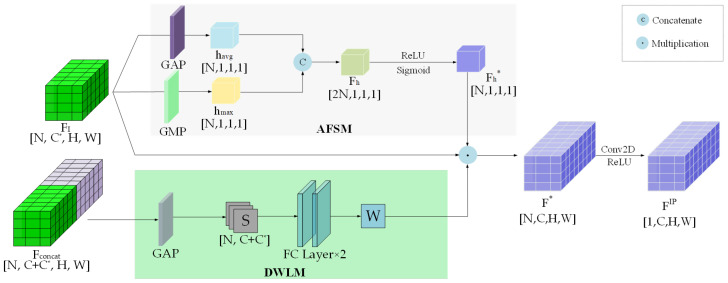
Adaptive feature fusion via channel and spatial weighting mechanisms. This framework uses global pooling to compress global information within the channel descriptors. We use global max pooling and global average pooling to extract the two channel descriptors ($h_{\max}\in R^{N\times 1\times 1\times 1}$ and $h_{\mathrm{avg}}\in R^{N\times 1\times 1\times 1}$). After concatenation to obtain the channel weights ($F_{h}\in R^{2N\times 1\times 1\times 1}$), we obtain the input channel descriptor weights ($F_{h}^{*}\in R^{N\times 1\times 1\times 1}$) through a linear layer with ReLU activation, where *N* is the maximum number of input CAVs (N is taken as 3). The learned channel feature weights are multiplied element-wise along the channel dimension with the features produced by the DWLM ($F_{\mathrm{concat}}$), resulting in a new feature representation ($F^{*}\in R^{N\times C\times H\times W}$). Finally, a 2D CNN with ReLU activation is applied to obtain the fused new feature ($F^{\mathrm{IP}}\in R^{1\times C\times H\times W}$).

During the feature fusion stage, the input is a 4D tensor ($F_{I}\in R^{N\times C^{*}\times H\times W}$). To extract the importance of each channel, the AFSM applies global average pooling and global max pooling to the input feature, thereby generating channel attention weights. The structure of the AFSM is illustrated in the upper part of Figure 4. The channel weights ($F_{h}^{*}\in R^{N\times 1\times 1\times 1}$) are applied to the input tensor ($F_{I}$) via channel-wise multiplication. The enhanced features are then linearly fused with DWLM-learned weights to form the fused feature ($F^{*}\in R^{N\times C\times H\times W}$). Subsequently, a 2D convolutional neural network (2D CNN) with channel compression is applied to refine the spatial dimensions and generate the final fused feature map ($F^{\mathrm{IP}}\in R^{1\times C\times H\times W}$). This operation preserves global information while standardizing the output dimensions, thereby improving the adaptability and efficiency of the network. The overall framework for multi-scale feature fusion is shown in Figure 4, and the complete multimodal fusion procedure is described in Algorithm 1. Finally, the fused feature map ($F^{\mathrm{IP}}$) is fed into an SSD detection head [41] to perform 3D object detection, including bounding-box localization and confidence score classification.
**Algorithm 1:** Adaptive Spatial and Channel Feature Fusion**Input**: Feature map $F_{I}\in R^{N\times C^{*}\times H\times W}$, $F_{\mathrm{concat}}\in R^{N\times (C+C^{*})\times H\times W}$**Output**: Fused feature $F^{IP}\in R^{1\times C\times H\times W}$AFSM: Channel Attention Branch      1. $h_{\mathrm{avg}}\leftarrow \mathrm{GAP}\left(F_{I}\right)$;      2. $h_{\max}\leftarrow \mathrm{GMP}\left(F_{I}\right)$;      3. $F_{h}\leftarrow \mathrm{Concat}\left(h_{\mathrm{avg}},h_{\max}\right)$;      4. $F_{h}^{*}\leftarrow \sigma \left(\mathrm{ReLU}\left(F_{h}\right)\right)$;      5. $F_{I}^{*}\in R^{N\times C\times H\times W}\leftarrow F_{I}⊙F_{h}^{*}$;      6. $F_{I}^{*}\in R^{1\times C\times H\times W}\leftarrow \mathrm{ReLU}\left(\mathrm{Conv}3D\left(F_{I}^{*}\right)\right)$;DWLM: Spatial Attention Branch      7. $s\leftarrow \mathrm{GAP}(F_{\mathrm{concat}})$;      8. $x\leftarrow \mathrm{ReLU}({\mathrm{FC}}_{1}\left(s\right))$;      9. $w\leftarrow \mathrm{Softmax}({\mathrm{FC}}_{2}\left(x\right))$;      10. w←reshape(w,N,C,1,1);      11. $F_{\mathrm{concat}}^{*}\in R^{N\times C\times H\times W}\leftarrow F_{\mathrm{concat}}⊙w$;Feature Fusion      12. $F^{*}\leftarrow F_{I}^{*}⊙F_{\mathrm{concat}}^{*}$;      13. $F^{IP}\leftarrow \mathrm{ReLU}\left(\mathrm{Conv}2D\left(F^{*}\right)\right)$;**return** $F^{IP}$;

### 3.5. Loss Function

The TS-IFF network proposed in this paper employs the loss function introduced in [27]. The total loss ($L_{\mathrm{total}}$) is composed of a classification loss and a regression loss:

(1) $$L_{\mathrm{total}}=\alpha L_{\mathrm{cls}}^{\mathrm{pos}}+\beta L_{\mathrm{cls}}^{\mathrm{neg}}+L_{\mathrm{reg}}$$

where α and β are positive constants that balance the relative importance and $L_{\mathrm{cls}}^{\mathrm{pos}}$ and $L_{\mathrm{cls}}^{\mathrm{neg}}$ denote the classification losses for positive and negative samples. The terms $L_{\mathrm{cls}}^{\mathrm{pos}}$ and $L_{\mathrm{cls}}^{\mathrm{neg}}$ are defined as follows:

(2) $$L_{\mathrm{cls}}^{\mathrm{pos}}=\frac{1}{N_{p}}\sum_{i=1}^{N_{p}}L_{\mathrm{cls}}\left(p_{i}^{\mathrm{pos}},1\right)$$

(3) $$L_{\mathrm{cls}}^{\mathrm{neg}}=\frac{1}{N_{n}}\sum_{j=1}^{N_{n}}L_{\mathrm{cls}}\left(p_{j}^{\mathrm{neg}},0\right)$$

where $p_{i}^{\mathrm{pos}}$ and $p_{j}^{\mathrm{neg}}$ are the softmax output probabilities for positive samples and negative samples, respectively. $N_{p}$ and $N_{n}$ denote the counts of positive and negative samples. $L_{reg}$ is the regression loss, which we define as follows:

(4) $$L_{reg}=\frac{1}{N_{p}}\sum_{i=1}^{N_{p}}L_{1}\left(u_{i}-{\hat{u}}_{i}\right)$$

where $u_{i}$ and ${\hat{u}}_{i}$ represent the regression ground truth and the predicted position, respectively, and $L_{1}$ denotes the smooth $L_{1}$ function:

(5) $$L_{1}\left(x\right)=\left\{\begin{array}{cc} \frac{x^{2}}{2} & \mathrm{if}\;|x|<1\\ \left|x\right|-\frac{1}{2} & \mathrm{if}\;x<-1\cup x>1 \end{array}\right.$$

## 4. Experimental Results

To evaluate the proposed model, we conducted targeted experiments separately on both simulated and real-world datasets: OPV2V [38] and V2V4Real [43] were utilized to assess cooperative perception capabilities, while CODD [44] was specifically used to evaluate performance in detecting small objects such as pedestrians. Additionally, extensive ablation studies and benchmark comparisons were carried out to demonstrate the superiority and effectiveness of the proposed cooperative perception model compared to existing state-of-the-art methods.

### 4.1. Datasets

The OPV2V dataset [38] is a simulation dataset that contains two subsets: Default Towns (DT) and Culver City (CC). The DT subset consists of data from eight default towns provided by CARLA [45] and contains on average about three CAVs per frame, with a minimum of two and a maximum of seven vehicles. The data in this subset was formally divided into a training set (6.7 K frames), a validation set (2 K frames), and a test set (2.7 K frames). The CC subset includes an independent test set of 550 frames to evaluate the model’s ability to generalize to new scenarios. All scenes last approximately 16.4 s and were captured using 64-channel LiDAR, generating approximately 1.3 million point clouds per second. This dataset simulates diverse urban driving conditions, including dynamic traffic flow, occlusions, and varying vehicle densities, providing a comprehensive benchmark for evaluating cooperative perception algorithms.

The CODD dataset [44] was also created with the help of the CARLA simulation platform. It contains 108 scene clips from eight different CARLA towns. For comparison with other methods, we use the same methodology as in [39]. Each scene consists of 125 frames, of which the first 100 frames are used for model training and the remaining 25 frames are used for testing. A notable feature of this dataset is that it includes varying numbers of vehicles and pedestrians, with the number of vehicles ranging from 4 to 15 and the number of pedestrians ranging from 2 to 8. CODD is the only collaborative sensing dataset that currently includes a pedestrian population. This diversity in participant types introduces additional complexity to the perception task, making it well-suited for evaluating models’ ability to detect and distinguish between heterogeneous traffic agents. Moreover, CODD provides detailed annotations for both vehicles and pedestrians, enabling fine-grained performance analysis across object categories and contributing to more realistic assessments of cooperative perception systems.

V2V4Real [43] is the first large-scale, publicly available real-world dataset for V2V cooperative perception, collected in Columbus, Ohio, across highways and urban streets. It includes 19 h of driving data with 310 K frames, from which 67 representative scenarios (10–20 s each) were selected. LiDAR and RGB frames were sampled at 10 Hz, yielding 20 K LiDAR point clouds and 40 K images. The dataset features high-density LiDAR point clouds and 240 K precisely annotated 3D bounding boxes for five classes. Sensor asynchronization between vehicles was kept below 50 ms. This dataset presents real-world challenges such as sensor noise, occlusion, and asynchronous multi-vehicle coordination, making it a valuable benchmark for validating the robustness and adaptability of cooperative perception models. Its diverse driving environments and dense traffic scenarios further enhance its utility for evaluating performance under complex real-world conditions.

### 4.2. Implementation Details

Our model was implemented with the PyTorch v1.7.1 framework [46] and trained and tested on a GeForce RTX 3090 GPU (NVIDIA Corporation, Santa Clara, CA, USA). The GPU has 24 GB RAM and runs in a CUDA v11.1 environment combined with cuDNN v8.0 for acceleration, ensuring efficient computation during inference. During the training process, the model uses a learning rate scheduler and an early stopping mechanism, and the optimizer was chosen to be Adam, with parameters set to ε=0.1 and a weight decay factor of $10^{-4}$. We trained the model for 30 epochs, and the model parameters were updated with a batch size of 2 and a learning rate of $2\times 10^{-3}$. The momentum was set to a value between 0.85 and 0.95. During the inference process, we filtered low-confidence bounding boxes with a threshold of 0.3 and used a non-maximum suppression strategy to remove overlapping candidates by setting the IoU threshold to 0.2.

The driving scenario was selected at any time during the following training process, and the number of CAVs was selected in the interval of [2,7], where the center vehicle is included in the interval as the EGO car (the car that receives all collaborative features). The number of CAVs was fixed for all scenes to ensure the fairness of the experiment. For data generation, we used the same parameters from [38,43] and set the range of LiDAR point clouds to [−3,1]×[−140.8,140.8]×[−40,40] meters as the range of z,x,y values for both OPV2V and V2V4Real. Similarly, for the CODD dataset, the range was set to [−6,4]×[−140.8,140.8]×[−40,40] meters. All datasets use the same body-column resolution of 0.4 m, which corresponds to a tensor size of [H×X]=[704×200] meters. For the SSD detection module, we used a vehicle anchor length, width, and height of [3.9,1.6,1.56] meters, a pedestrian anchor length, width and height of [0.6,0.6,1.7] meters and an anchor-box rotation angle range of [0,90] degrees.

In autonomous driving 3D point-cloud feature fusion experiments, we utilized AP metrics to provide a comprehensive evaluation of detection performance. AP captures the balance between precision and recall across varying confidence thresholds. Specifically, AP0.5 and AP0.7 correspond to the average precision computed at IoU thresholds of 0.5 and 0.7, respectively, which are commonly used to assess the detection accuracy of larger objects such as vehicles. For smaller or more variable targets like pedestrians, we adopted a lower IoU threshold, AP0.1, to more appropriately evaluate the model’s detection capabilities. By incorporating AP metrics at different IoU thresholds, we achieved a more thorough and nuanced assessment of the model’s effectiveness across diverse object categories and scales, thereby offering deeper insights into its strengths and limitations.

### 4.3. Experimental Results

Table 1 shows the quantitative experimental results of our proposed model on the simulated OPV2V and CODD datasets. Using the detection of individual vehicles without collaborative sensing as a baseline, we benchmark our model against the SOTA methods. Of the state-of-the-art methods listed in the table, DiscoNet, CoBEVT, and HM-ViT are specifically designed for the features and scenarios of the DT sub-dataset and the V2X-Sim dataset. In contrast, the CC sub-dataset and CODD datasets contain more diverse transformations, complex scenarios, and small targets, which fall outside the optimal application conditions for these methods. In summary, the comparison methods selected in this work are representative and closely related to our task. They are evaluated on similar datasets and metrics to ensure a fair comparison. Their implementations and results are publicly available, supporting reproducibility and meaningful benchmarking.

The experimental results show that our model achieves an improvement in AP of up to 65% over the baseline. In particular, for object detection of surrounding vehicles, our model shows an AP improvement of about 2% to 4% over the SOTA for both the OPV2V and CODD datasets. In particular, for the DT and CC subsets of the OPV2V dataset, our model achieves a detection accuracy of over 90% for both the AP@0.5 and AP@0.7 thresholds. For small-object pedestrian detection within the CODD dataset, our model achieves an accuracy of over 60%, which is a significant increase of 29.2% AP compared to the state-of-the-art best method. Moreover, to better adapt to the complexity of the real world and enhance perception consistency and decision reliability, we conducted model testing on the real-world V2V4Real dataset. As shown in Table 2, our method achieved AP values of 68.2% and 40.1% at thresholds of 0.5 and 0.7, respectively, for vehicle detection, outperforming other methods. Compared to the second best approach, our model demonstrated a performance improvement of 2.5% to 8% across different thresholds, further demonstrating the model’s outstanding performance and clear advantages in detecting surrounding vehicles in cooperative perception tasks.

**Figure 5:**
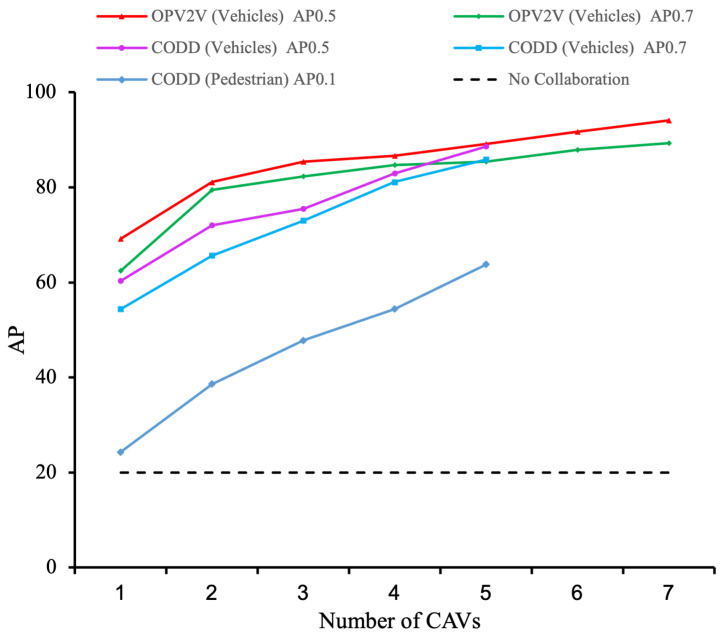
Effect of CAV numbers on the accuracy of cooperative perception: curves at different thresholds in the DT and CODD datasets (viewing in color is recommended for clarity).

**Figure 6:**
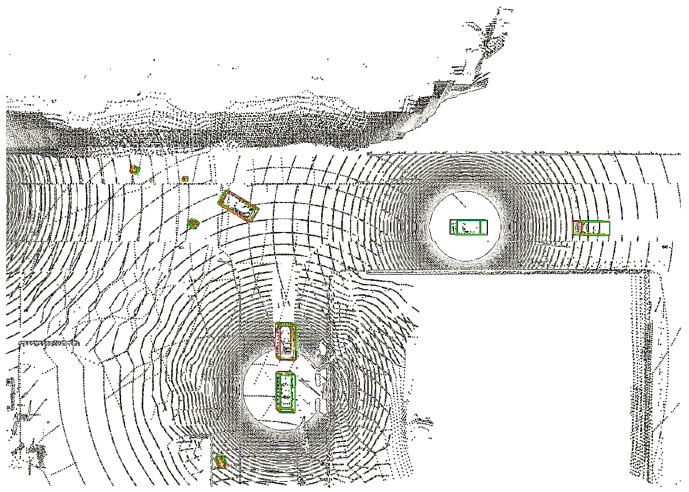
Visualization of the results of the TS-IFF model on the CODD dataset. The figure shows a driving scene with a blind spot where the $CAR_{ego}$ is able to accurately detect pedestrians and other objects outside its field of view through collaborative perception with $CAR_{1}$. Ground truth (GT) is denoted by green rectangles, while predictions are shown in red.

The results presented in Figure 5 show the evident trend in collaborative perception: as the number of CAVs in the collaborative perception network increases (up to seven CAVs in the OPV2V dataset and five CAVs in the CODD dataset), there is a significant improvement in detection performance. The vehicle detection accuracy (AP@0.5) improved by 26.5% and 31.9% in the two datasets, respectively, while the pedestrian detection accuracy (AP@0.1) increased by 61.9% in the CODD dataset. Meanwhile, we also conducted extensive experiments on the CODD dataset, which contains more pedestrians. The qualitative results are shown in Figure 6, demonstrating that in driving scenarios with blind spots, we can successfully detect pedestrians through collaborative perception. These results show that the detection of small objects benefits significantly from collaborative perception and that our proposed method significantly improves the detection of these small objects.

**Figure 7:**
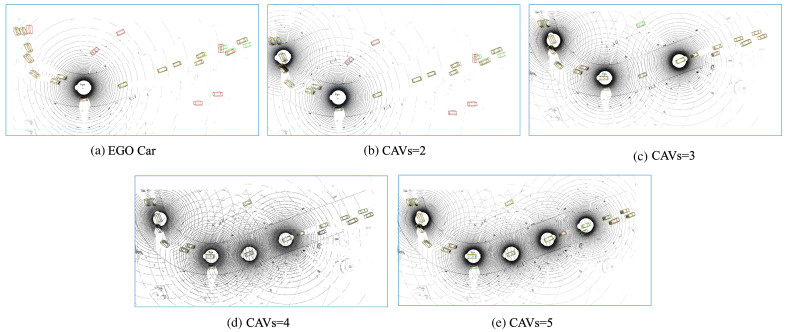
TS-IFF visualization of the effects of collaborative perception as the number of CAVs increases. The figure shows the prediction results for the DT sub-dataset in subfigures (**a**–**e**). Ground truth (GT) is denoted by green rectangles, while predictions are shown in red. These images are best viewed in color.

**Figure 8:**
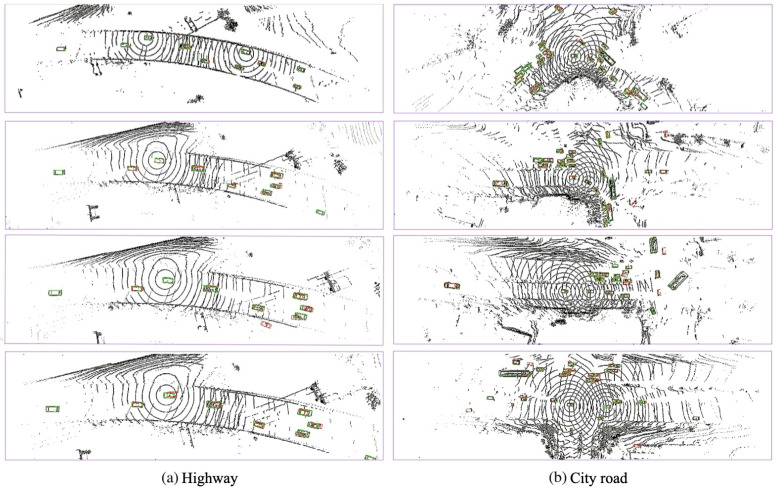
TS-IFF visualization results in two real-world scenarios. The ground truth (GT) is represented by green rectangles, while predictions are indicated by red rectangles. The correspondence between the GT and predictions is highlighted with yellow rectangles. These images are best viewed in color.

Figure 7 and Figure 8 present the qualitative visualization results on the simulated DT and CODD simulated, as well as the V2V4Real dataset, showcasing multi-vehicle collaborative perception in simulated driving scenarios. As observed in Figure 7, when relying solely on a single central vehicle (without fusion), certain objects in the scene may be misidentified as vehicles, while some distant targets may be entirely missed due to occlusion. As more collaborative autonomous vehicles (CAVs) participate in cooperative perception, the central vehicle gains an expanded field of view and richer sensor data, enabling more accurate and reliable detection of distant objects while reducing false positives and missed detections. Consequently, our model demonstrates outstanding robustness in both simulated and real-world scenarios.

Additionally, in the simulated OPV2V dataset (Figure 7a) includes typical qualitative examples where sparse LiDAR inputs lead to occasional false positives and a slight degradation in detection performance. While the model generally performs well in identifying vehicle targets, in scenarios with extreme sparsity or missing information, some non-vehicle objects may be mistakenly classified as vehicles, and the detection accuracy for distant or occluded targets is somewhat reduced. This highlights the importance of multi-sensor fusion and collaborative perception in enhancing the comprehensiveness of scene understanding. In the analysis of the real-world V2V4Real dataset, visualization results reveal that factors such as occlusion, sparse object distribution, and sensor noise in real environments can still affect detection outcomes. Occlusion causes partial loss of point-cloud information in certain areas, increasing the difficulty of accurate recognition, while sensor noise may lead to occasional false detections or uncertainties.

### 4.4. Ablation Study

To evaluate the impact of the proposed AFSM and DWLM on 3D object detection performance, we conducted a series of ablation experiments with seven CAVs for the OPV2V dataset and three CAVs for the CODD dataset. In the baseline setup, we excluded the AFSM and DWLM and directly fused the intermediate-level features via simple concatenation without generating pseudo-images. We then incrementally activated each module (DWLM and AFSM) to evaluate their individual contributions. All experiments were performed using an SSD detection head. The results, summarized in Table 3, indicate significant performance improvements when the DWLM and AFSM are integrated. In particular, for the OPV2V dataset, vehicle detection accuracy increased by 9% at AP@0.5, while pedestrian detection accuracy improved by 18.5% at AP@0.1 for the CODD dataset. We provide further statistics on the model’s inference time compared to baseline methods, demonstrating that the proposed approach achieves notable performance gains while keeping computational overhead within a practical and acceptable range, supporting its feasibility for real-world deployment.

In a subsequent ablation study, we investigated the impact of fusing pseudo-images with different resolutions on 3D object detection in the environment. Consistent with previous experiments, we used seven CAVs for the OPV2V dataset as the upper bound for the ablation study and three CAVs for the CODD dataset. The results presented in Table 4 show that in the OPV2V dataset, the fusion of intermediate features with pseudo-images achieves an average precision of 94.1% for vehicle detection at AP@0.5, representing an improvement of 17.4%. For the CODD dataset, which focuses specifically on pedestrian detection, the fusion of intermediate features with pseudo-images achieves an average precision of 69.1% for small-object detection (e.g., pedestrians) at AP@0.1—nearly a 40% improvement compared to the baseline experiment.

Overall, the results presented in Table 4 indicate that increasing the resolution of pseudo-images consistently improves detection accuracy, especially for small objects such as pedestrians. However, the performance gains tend to plateau after reaching a certain resolution, with diminishing returns and a limited impact on overall perception performance from further increases. Therefore, in practical applications, selecting an appropriate resolution is crucial to achieving optimal system performance.

**Figure 9:**
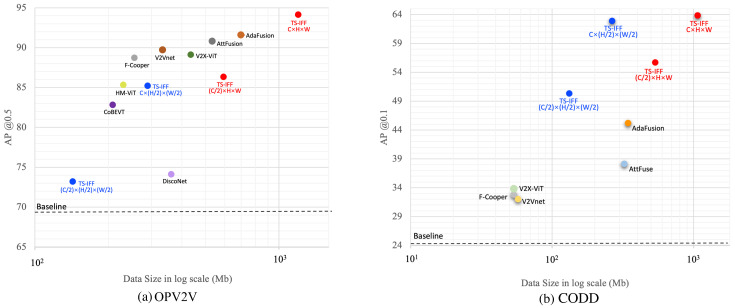
Ablation results showing the relationship between the performance and bandwidth of the latest models on two datasets. (**a**) Results on OPV2V. (**b**) Results on CODD. Red and blue points represent **TS-IFF** performance under different resolutions. Best viewed in color.

In V2V collaborative perception networks, communication bandwidth serves as a vital factor that directly influences both the speed and efficiency of information transmission between connected autonomous vehicles. To thoroughly assess the performance of our proposed method in terms of network communication bandwidth consumption, we conducted a series of detailed ablation experiments using two representative simulated datasets. These experiments aimed to explore and analyze the relationship between detection performance and bandwidth requirements. The results are presented in Figure 9a. It is evident that our method achieves the highest target recognition accuracy, though this comes with relatively high bandwidth use. However, as the feature resolution decreases, an inevitable but acceptable decline in AP is observed. We speculate that more aggressive downsampling leads to greater loss of keypoint information, reducing recognition accuracy. From the perspective of collaborative perception, the trade-off between performance and bandwidth, as shown in Figure 9b, is reasonable.

Moreover, in the small-target pedestrian recognition experiment, when we reduced the number of feature channels and the resolution to half of the original values, the resulting detection accuracy and bandwidth overhead reached an optimal balance. This also suggests that even with simple downsampling for feature compression, our model can still maintain optimal recognition accuracy for small-target detection.

## 5. Conclusions

In this paper, we introduce a novel perception architecture, TS-IFF, which integrates multiple feature types to improve the effectiveness of collaborative perception. Specifically, we propose a two-stage intermediate feature fusion strategy that optimizes and integrates intermediate features across different levels to enhance perception performance. Additionally, we designed a feature weight learning mechanism to adaptively fuse high-resolution pseudo-images with intermediate features. Pseudo-images preserve the spatial structure and geometry of point clouds, while intermediate features capture multi-scale contextual semantics at multiple levels. Experimental results demonstrate that the TS-IFF model excels in detecting small 3D objects, such as pedestrians, while maintaining lightweight bandwidth requirements. This effectively addresses the limitations of traditional non-fusion methods under occlusion and bandwidth constraints.

While our method achieves a good balance between perception accuracy and communication efficiency, its performance in extremely complex urban scenarios still faces robustness challenges. In future work, we plan to further reduce bandwidth consumption and improve system robustness by developing a more efficient autoencoder-based encoding and decoding mechanism, enabling optimal compression of features while preserving critical perception performance.

## Figures and Tables

**Table 1:** Quantitative comparison of the TS-IFF model with state-of-the-art methods across two datasets. Bold highlights denote best performance, with blue values in parentheses indicating AP improvement over the second best method and red values indicating parameter increases compared to this method. Underlined values indicate second best results. ↑: Larger values are better. ↓: Smaller values are better.

Method	OPV2V				CODD			Para (M) Total (↓)
	DT		CC		Vehicle		Pedestrian	
	AP@0.5	AP@0.7	AP@0.5	AP@0.7 (↑)	AP@0.5	AP@0.7	AP@0.1 (↑)	
Baseline	69.2	62.4	55.3	47.6	60.3	54.4	24.3	**6.58**
F-Cooper [33]	88.7	79.0	84.6	72.8	77.6	74.3	32.8	7.27
V2VNet [47]	89.7	82.2	86.0	73.4	80.3	75.8	32.0	14.61
AttFuse [38]	90.8	81.5	85.4	73.5	81.4	77.7	38.1	**6.58**
DiscoNet [48]	74.1	59.0	–	–	–	–	–	9.66
CoBEVT [36]	82.8	63.7	–	–	–	–	–	8.35
V2X-ViT [35]	89.1	82.6	87.3	73.7	82.3	78.9	33.8	13.45
HM-ViT [49]	85.3	76.3	–	–	–	–	–	17.64
AdaFusion [39]	91.6	85.6	88.1	79.0	86.2	83.9	45.2	7.27
**Ours**	**94.1 (+2.7%)**	**89.3 (+4.1%)**	**90.3 (+2.4%)**	**82.1 (+3.8%)**	**88.6 (+2.7%)**	**85.8 (+2.2%)**	**63.8 (+29.2%)**	8.16 (+10.9%)

**Table 2:** Comparison of the TS-IFF model with SOTA methods for vehicle detection on the V2V4Real dataset. Bold highlights indicate the best performance, with blue values in parentheses representing the accuracy improvement over the second best method. Underlined values indicate the second best results.

Method	V2V4Real	
	AP@0.5	AP@0.7
Baseline	39.8	22.0
F-Cooper	60.7	31.8
V2Vnet	64.5	34.3
AttFuse	64.7	33.6
V2X-ViT	64.9	36.9
CoBEVT	66.5	36.0
**Ours**	**68.2 (+2.5%)**	**40.1 (+8.0%)**

**Table 3:** Ablation study to investigate the impact of the proposed modules (AFSM and DWLM) on network performance without the fusion of pseudo-images. Baseline represents collaborative results without these modules. Best results are highlighted in bold, ↑: Larger values are better. ↓: Smaller values are better.

Model	AFSM	DWLM	OPV2V (AP@0.5↑)	CODD (AP@0.1↑)	Para. (M)	Ave Infer. Time (ms/Frame)
Baseline	×	×	69.8	24.7	6.58	15.63
	×	✓	70.2	26.6	7.03	26.18
TS-IFF	✓	×	72.1	27.8	7.27	27.33
	✓	✓	**76.7**	**30.3**	8.16	29.60

**Table 4:** Ablation study exploring the impact of intermediate feature resolution. Baseline represents collaborative results without fusing pseudo-images. The **PI** column represents the use of pseudo-images and their resolution with respect to intermediate features. The best results in each setting are clearly emphasized in bold for comparison.

Model	PI (C × H × W)	OPV2V (AP@0.5↑)	CODD (AP@0.1↑)
Baseline	–	76.7	30.3
TS-IFF	C×(H/2)×(W/2)	85.2	62.9
	(C/2)×(H/2)×(W/2)	73.2	50.3
	C×H×W	**94.1**	65.9
	(C/2)×H×W	86.3	55.7
	C×(2H)×(2W)	90.7	**69.1**

## Data Availability

Data available on request due to privacy restrictions.

## Abbreviations

The following abbreviations are used in this manuscript:

V2V	Vehicle to vehicle
AVs	Autonomous vehicles
CAVs	Connected autonomous vehicles
TS-IFF	Two-stage intermediate-level feature fusion
DWLM	Dynamic weight learning mechanism
AFSM	Adaptive feature selection module
SSD	Single-shot detector
FPN	Feature pyramid network
VFE	Voxel feature encoding
BEV	Bird’s-eye view
3D CNN	3D convolutional neural network
2D CNN	2D convolutional neural network
DT	Default Towns
CC	Culver City
GT	Ground truth
PI	Pseudo-image
